# Study on the Anti-Icing and De-Icing Performance of a New Superhydrophobic Coating PTFE/SiO_2_-ER/FR Composite

**DOI:** 10.3390/ma19112352

**Published:** 2026-06-02

**Authors:** Xinggui Lei, Shifeng Liu, Qiuyan Xie, Yue Zhang, Binni Zou, Yuan Yuan

**Affiliations:** 1 College of Materials Science and Engineering, Chongqing University, Chongqing 400044, China; leixinggui202403@163.com (X.L.); 19942362536@163.com (Q.X.); zyfrost@163.com (Y.Z.); yolanda@stu.cqu.edu.cn (B.Z.); 2 Electron Microscopy Center of Chongqing University, Chongqing University, Chongqing 400044, China

**Keywords:** superhydrophobic coating, PTFE/SiO_2_-ER/FR composite, spraying method, anti-icing/de-icing performance, anti-icing/de-icing durability

## Abstract

**Highlights:**

- PTFE/SiO_2_–ER/FR superhydrophobic coatings for anti-icing and de-icing of insulators were designed.
- The micro/nanoscale hierarchical structure imparts good superhydrophobicity to PTFE/SiO_2_–ER/FR.
- PTFE/SiO_2_–ER/FR superhydrophobic coatings achieve good durability against UV irradiation or freeze–thaw cycling.
- PTFE/SiO_2_–ER/FR superhydrophobic coatings possess good anti-icing and de-icing performance under natural icing conditions.

**Abstract:**

This work describes the preparation of PTFE (polytetrafluoroethylene)/SiO_2_ (silicon dioxide)–ER (epoxy resin)/FR (fluorosilicone resin) superhydrophobic coatings using the spray method to improve the anti-icing and de-icing performance of transmission line insulators. The coatings exhibit a consistent fluorine distribution (32.86 wt%), which enhances their low surface energy, alongside SiO_2_ nanoparticles that occupy the interstices between PTFE particles, resulting in a dense micro- and nanoscale hierarchical structure. Consequently, the coatings have good superhydrophobicity, featuring a contact angle of 173.9° and roll angle of 1.2°. Following 66 days of UV irradiation, the contact angle remains above 150°, and the roll angle is approximately 15°, accompanied by a slight increase in ice adhesion strength. Following 26 freeze–thaw cycles, the contact angle stabilizes at around 157°, showing good environmental durability. Natural icing studies validate the coatings’ good anti-icing and de-icing efficacy: in comparison to common insulators, the coated insulators demonstrate a 14.2% reduction in ice accretion weight and a 67.7% reduction in maximum ice ridge length.

## 1. Introduction

Ice accumulation on transmission lines can result in structural damage, thereby interrupting the power supply to residents [1]. In February 2021, a winter storm named Uri struck Texas, USA, where extreme freezing temperatures caused power grid failures and transmission line disruptions, leaving millions of residents without electricity [2]. In early 2018, approximately 1200 transmission lines in Jiangxi Province, China, suffered from icing-induced failures under severe rain and snow conditions, resulting in power outages affecting more than 820,000 households [3]. Ice bridging between adjacent insulators in insulator strings on transmission lines, combined with the conductive performance of contaminants contained in the melted ice film, is one of the potential factors that can trigger ice flashovers and tripping, which in turn can lead to widespread power outages [4,5]. Therefore, effective anti-icing and de-icing strategies for transmission line insulators are essential to ensure the safe and stable operation of transmission lines in winter.

When the ambient temperature falls below 0 °C, supercooled water droplets condense and freeze on transmission lines. Measures adopted to prevent ice formation are defined as anti-icing. Conversely, de-icing pertains to the elimination of ice that has already accumulated on transmission lines. Anti-icing and de-icing strategies are generally categorized into two principal types: active and passive solutions. Active solutions rely on external energy input or mechanical forces to melt ice deposits or induce fracture and shedding. Thermal de-icing [6] and mechanical de-icing [7] are among the common technologies for active solutions. Passive solutions reduce or obstruct ice adhesion without relying on external energy sources or mechanical force [8]. According to references [9,10], superhydrophobic coatings are reported as effective passive solutions that can delay the freezing process, reduce ice adhesion, and facilitate the shedding of accumulated ice under external wind or temperature variation. Therefore, superhydrophobic coatings have garnered considerable interest due to their performance.

The combination of low-surface-energy chemical modification and micro- and nanoscale rough structures forms the basis of the preparation of superhydrophobic coatings. Fang et al. [11] fabricated a superhydrophobic poly(vinylidene fluoride) (PVDF) coating through an ice-template method, which created micro- and nanoscale rough structures without additional low-surface-energy modification. Kavya et al. [12] developed a robust superhydrophobic coating using a sol–gel process, which maintained its water repellency after 200 h of weathering and 16 de-icing cycles. Fu et al. [13] employed a composite electrodeposition method to construct micro- and nanoscale rough structures together with in situ low-surface-energy modification, producing a robust superhydrophobic coating with good anti-icing and de-icing performance. Wang et al. [14] used a plasma-enhanced chemical vapor deposition (PECVD) method to construct micro- and nanoscale rough structures together with low-surface-energy silica chemistry, successfully producing a uniform superhydrophobic coating on the inner surface of tubes. Li et al. [15] reported a layer-by-layer self-assembly strategy to construct hierarchical micro- and nanoscale structures with low surface energy, producing a robust superhydrophobic coating for efficient oil-water separation. Guo et al. [16] demonstrated a spraying method to construct hierarchical micro- and nanoscale structures with low-surface-energy chemistry, producing a robust superhydrophobic coating with good mechanical and anti-corrosion performance. Zeng et al. [17] employed a spraying method to construct hierarchical micro- and nanoscale structures together with low-surface-energy siloxane chemistry, producing a superhydrophobic coating with good mechanical durability and anti-corrosion performance. Among the previously described preparation techniques, the spray-coating method is both pragmatic and appropriate. This method maintains the microstructure and chemical characteristics of the raw materials, enables largescale industrial production of superhydrophobic coatings, and is appropriate for uniform film application on surfaces with intricate geometries [18,19].

Nevertheless, some studies have indicated that the long-term durability of superhydrophobic coatings for anti-icing and de-icing performance is insufficient [20,21]. Researchers have examined a variety of materials to address this issue. Polytetrafluoroethylene (PTFE) is utilized as a low-surface-energy material [22,23], whereas silicon dioxide (SiO_2_) nanomaterials are frequently employed to create microscale rough structures [24]. The integration of these two materials facilitates the preparation of superhydrophobic coatings exhibiting good anti-icing and de-icing performance, characterized by a high contact angle, notably delayed ice nucleation, and substantially reduced ice adhesion strength [25]. Epoxy resin (ER) comprises low-molecular-weight prepolymers with numerous epoxy groups, distinguished by its ease of production, high safety, and good adhesive characteristics [26]. Moreover, ER serves as a good electrical insulator, safeguarding electrical components from short circuits, dust, and moisture [27]. The molecular composition of fluorosilicone resin (FR) comprises fluorocarbon chains and C-F bonds, which inherently confer high bond energy and low surface free energy [28]. Furthermore, heptadecafluorodecyltrimethoxysilane (FAS-17) is utilized for the hydrophobic modification of SiO_2_. The fluorinated chain can be attached to the silica surface, hence providing low surface energy for superhydrophobic coatings [29]. Consequently, the use of these materials is anticipated to further reduce the surface energy of the coating, which is expected to improve weather resistance and maintain hydrophobicity to a certain extent.

The aim of this study was to develop PTFE/SiO_2_–ER/FR superhydrophobic composite coatings utilizing a spray-coating method. In the coatings, PTFE and SiO_2_ function as composite nanofillers, ER and FR act as composite binders, and FAS-17 serves as a low-surface-energy modifier. The anti-icing and de-icing performance of the PTFE/SiO_2_–ER/FR composite coatings was examined, focusing specifically on their efficacy in natural field conditions.

## 2. Experimental Section

### 2.1. Preparation of PTFE/SiO_2_-ER/FR Dispersed Solution for Subsequent Spraying

An analytical balance (FA1004N, Shanghai Precision Instrument Co., Ltd., Shanghai, China) was utilized to measure 100 g of analytical-grade ethyl acetate (Chongqing Chuandong Chemical Co., Ltd., Chongqing, China) and 1 g of analytical-grade FAS-17 (Shanghai Aladdin Biochemical Technology Co., Ltd., Shanghai, China). PTFE particles (approximately 200 nm, Shanghai McLean Biochemical Technology Co., Ltd., Shanghai, China) and SiO_2_ nanoparticles (7–40 nm, Shanghai McLean Biochemical Technology Co., Ltd., Shanghai, China) were measured according to the predetermined mass ratio, ensuring that the total mass of the two fillers was maintained at 4.3 g, with SiO_2_ accounting for about 25% by mass. All aforementioned raw materials were combined in a beaker, magnetically agitated at 500 rpm using a magnetic stirrer (ZNCL-BS, Chongqing Dongyue Instrument Co., Ltd., Chongqing, China) for 20 min, followed by ultrasonic treatment for 10 min in an ultrasonic cleaner (DY-6.5-180DG, Chongqing Dongyue Instrument Co., Ltd., Chongqing, China). Thereafter, industrial-grade ER (Kunshan Jiulimei Electronic Materials Co., Ltd., Kunshan, China) and FR (F content ≥ 26.0 wt%, Chengdu Aikeda Chemical Technology Co., Ltd., Chengdu, China) were measured, totaling 8 g (ER: 6.8 g, FR: 1.2 g), followed by magnetic stirring for 20 min. Subsequently, an industrial-grade ER curing agent (Kunshan Jiulimei Electronic Materials Co., Ltd., Kunshan, China) was incorporated in a mass ratio of 10:3 (ER: curing agent) and subjected to magnetic stirring for 5 min, yielding a homogeneously dispersed coating solution.

### 2.2. Preparation of PTFE/SiO_2_-ER/FR Composite Coatings on Glass Slides and Glass Insulators

**Figure 1:**
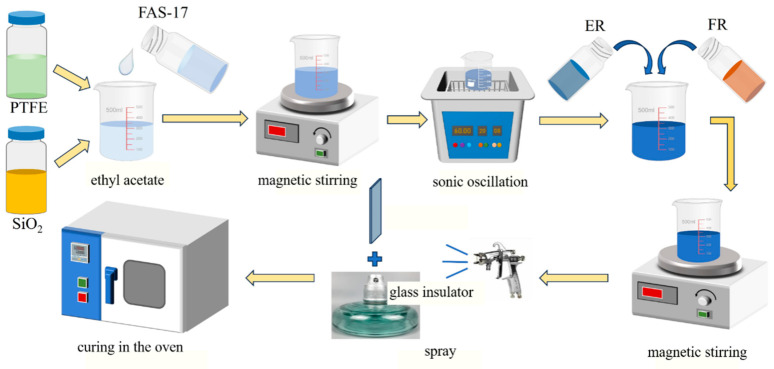
The preparation process for the PTFE/SiO_2_-ER/FR superhydrophobic coatings.

To assess the anti-icing and de-icing efficacy of PTFE/SiO_2_–ER/FR composite coatings in both laboratory and natural conditions, glass slides and glass insulators were chosen as representative substrates for subsequent sample processing (Figure 1). Glass slides (76.2 mm × 25.4 mm × 1 mm) and FC70/146-type glass insulators were consecutively cleaned with anhydrous ethanol and deionized water generated using a deionization system (ATSrese1610A, Chongqing Anderson Instrument Co., Ltd., Chongqing, China). Subsequent to drying, all substrates were positioned in a vacuum drying oven (DZF-9076A, Shanghai Jinghong Instrument Co., Ltd., Shanghai, China) and subjected to a temperature of 70 °C for a duration of 5 min. The pre-cleaned glass slides were affixed in a fixture, and the glass insulators were positioned on a constant-speed rotating platform. The prepared coating solution was uniformly applied to the substrate surface using an electric spray gun (ZP-PQQ-001, Zhipu Tools International Group Co., Ltd., Hong Kong, China; nozzle diameter 1 mm). The optimal spraying parameters were established using a single-factor experiment, with the following specifications: spraying distance of 30 cm, spraying duration of 12 s, and liquid flow rate of 60 mL/min. Following initial drying at ambient temperature, the sprayed samples were relocated to the previously stated vacuum oven and subjected to heat curing at 70 °C for 4 h. In later experiments, coated glass slides were employed for laboratory performance evaluation, whilst coated glass insulators were utilized for field tests in natural icing conditions.

### 2.3. Microstructure Characterization of Coatings

The coatings’ surface microstructure was analyzed utilizing a field-emission scanning electron microscope (FESEM, Auriga, Zeiss, Oberkochen, Germany). An energy-dispersive X-ray spectrometer (EDS) integrated into the FESEM system was utilized for elemental composition investigation. Before characterization, all samples were sputter-coated with a thin layer of gold to improve surface conductivity. FESEM imaging was executed at an acceleration voltage of 3–5 kV, whereas EDS elemental analysis was carried out at 20 kV.

### 2.4. Durability Performance Test Experimental Conditions

The glass slides coated with PTFE/SiO_2_–ER/FR, measuring 76.2 mm × 25.4 mm × 1 mm, were subjected to ultraviolet (UV) radiation damage testing in a UV accelerated weathering chamber. A Q-LAB340 UV lamp (Q-LAB Corporation, Westlake, OH, USA) was utilized, featuring an emission wavelength range of 315 to 400 nm and a peak irradiance of 1.2 W/m^2^ at 340 nm. The test conditions included a 6 cm distance between the sample and the lamp, a temperature of 30 °C, and a duration of 66 days. The static contact angle and roll angle of the coating were assessed every two days during the test. The ice adhesion strength of the coating was assessed following UV radiation. The coating was analyzed using SEM and EDS to examine the impact of UV radiation on its surface microstructure and elemental composition.

During the freeze–thaw cycle test, the superhydrophobic coating samples were situated on a cooling platform, with an ice-forming mold placed on the coating surface. One milliliter of deionized water was introduced into the mold, the cooling platform was engaged, and the temperature was adjusted to −10 °C. After the water in the mold had entirely frozen, the cooling platform was deactivated to permit the ice to melt naturally, thus concluding one freeze–thaw cycle. The contact angle and roll angle of the samples were assessed following every two freeze–thaw cycles. Following the testing, the ice adhesion strength of the coating was assessed, and the coating was analyzed using SEM and EDS to examine alterations in its surface microstructure and elemental composition.

Each group underwent testing with a minimum of three parallel samples, and all tests were conducted in triplicate. Data were expressed as mean ± standard deviation (SD).

### 2.5. Measurement of Contact Angle, Roll Angle and Ice Adhesion Strength

The contact angle of water was assessed using an SDC-100 contact angle measuring device (Dongguan Shengding Precision Instrument Co., Ltd., Dongguan, China) with an accuracy of ±0.1°. Measurements were performed at ambient temperature (26 °C) and 55% relative humidity (RH). A 5 μL droplet of deionized water was administered to the sample surface using a micro-syringe (Dongguan Shengding Precision Instrument Co., Ltd., Dongguan, China). Upon stabilization of the droplet, the contact angle was determined using semi-automatic fitting with the elliptical differential algorithm integrated into the accompanying program. To guarantee test reliability and data precision, five random spots were chosen on each sample for evaluation, and the final contact angle was calculated as the mean of the five measurements.

The specimen was affixed to the tilting apparatus included with the SDC-100 contact angle measurement device (Dongguan Shengding Precision Instrument Co., Ltd., Dongguan, China). A 5 μL droplet of deionized water was applied to the sample surface using a micropipette (Dongguan Shengding Precision Instrument Co., Ltd., Dongguan, China). After the droplet stabilized, the tilt angle of the test platform was gradually increased while continuous observation was conducted. The tilt angle at which the droplet commenced sliding was recorded as the roll angle. Five independent measurements were conducted for each sample, and the arithmetic mean was calculated as the final result. The testing conditions were set at room temperature (26 °C) and 55% RH.

The coated specimen was positioned on a temperature-regulated cooling platform calibrated to −10 °C, maintaining an ambient humidity of 55% RH. A PTFE cylindrical mold with a diameter of 1.8 cm was placed on the coated surface, and 1 mL of deionized water was introduced into the mold. The cooling platform was then activated to initiate the freezing process. After the water in the mold had fully frozen, a force gauge was employed to exert a shear force at a constant rate, progressively increasing the force until the ice layer separated from the coated surface. The maximum shear force at interface separation was recorded. The test was conducted at multiple locations on each sample, and the mean ice adhesion strength was calculated.

### 2.6. Water Droplet Bouncing Test 

The device employed for high-speed imaging of water droplet dynamics was the Qianyanlang M220 high-speed camera (Hefei Zhongke Junda Vision Technology Co., Ltd., Hefei, China) (maximum resolution 800 × 600, full-frame frame rate 2000 fps, minimum exposure duration 1 μs). In single-droplet impact experiments, a 34G fine needle was employed to dispense a deionized water droplet, approximately 2 mm in diameter, onto the sample surface from a height of 10 cm. Atomized droplets were administered through a fine-orifice spray bottle. The high-speed camera documented the bouncing behavior of water droplets on superhydrophobic-coated and uncoated glass samples in both controlled and natural settings. A fiber-optic cold light source (Olympus LG-PS2, Olympus Corporation, Tokyo, Japan) was utilized for additional illumination during filming.

### 2.7. Natural Icing Observation Test Under Field Conditions

Natural icing experiments on superhydrophobic-coated specimens were performed at the Meihua Mountain Anti-icing Base of China Southern Power Grid. During the experiments, the mean ambient temperature was −4 °C, relative humidity was sustained at 90–100% RH, and wind velocities fluctuated between 0 and 5 m/s. Surface wettability was assessed utilizing an SDC-30 portable contact angle measurement device (Dongguan Shengding Precision Instrument Co., Ltd., Dongguan, China). Under natural icing conditions, the contact angles and roll angles of both superhydrophobic and common insulators were assessed.

The in situ droplet bounce behavior on the surfaces of both types of insulators was examined in the field icing environment, and the dynamic bounce process of water droplets on the coated surfaces was evaluated. Field tests for natural icing on superhydrophobic and common insulators were performed using a 4 m × 4 m × 2 m icing observation frame, with a cumulative test period of 166 h. A load cell was employed to continuously monitor the sample mass. Photographs of the macroscopic icing morphology were captured for all insulators at 20 h intervals, while the icing mass was concurrently documented.

All tests were conducted on a minimum of three separately prepared samples, with each test replicated three times to ensure repeatability.

## 3. Results and Discussion

### 3.1. Surface Micro/Nanostructure Characterization of PTFE/SiO_2_-ER/FR Composite Coatings

**Figure 2:**
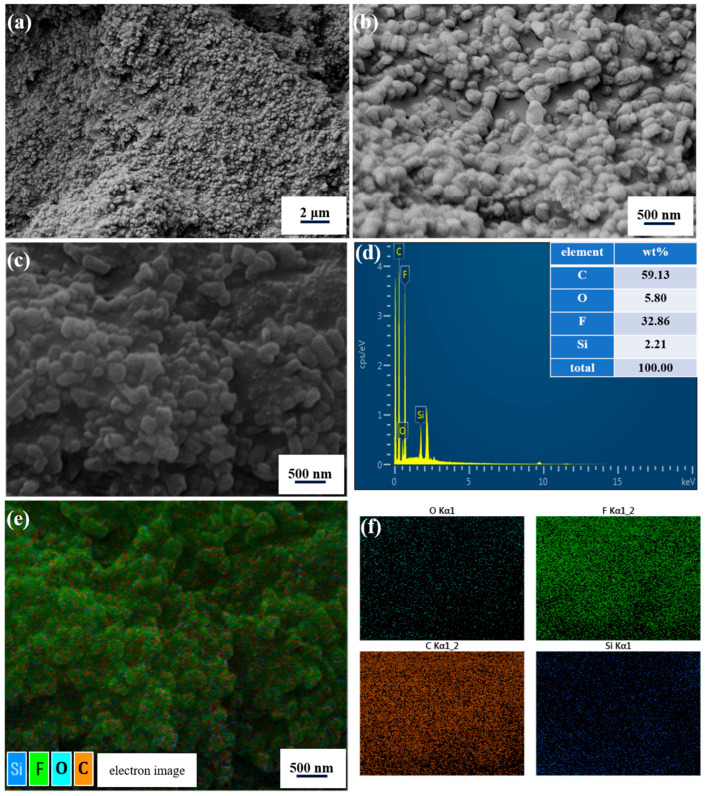
SEM and EDS images of the PTFE/SiO_2_–ER/FR composite coatings. (**a**–**c**) SEM images at different magnifications; (**d**) EDS spectrum and elemental composition; (**e**,**f**) elemental mapping of Si, F, O, and C.

To verify the successful preparation of superhydrophobic coatings, SEM and EDS characterizations were performed on the PTFE/SiO_2_–ER/FR composite coatings (Figure 2). The low-magnification SEM image (Figure 2a) illustrates that the coated surface is uniformly adorned with densely packed micro- and nanoparticles, exhibiting no discernible faults or fissures. High-magnification SEM images (Figure 2b,c) elucidate a characteristic micro–nano hierarchical roughened structure: larger PTFE microparticles constitute the primary micron-scale framework, whereas smaller SiO_2_ nanoparticles are uniformly distributed, occupying the interstitial voids and resulting in a dense and uniform roughened structure. The micro–nano architecture is essential for attaining stable superhydrophobicity and smaller ice adhesion [30,31,32].

The EDS spectra (Figure 2d) validates the existence of all target elements: C (59.13 wt%), O (5.80 wt%), F (32.86 wt%), and Si (2.21 wt%). The elevated fluorine concentration (32.86 wt%) may signify a prevalence of C–F bonds originating from PTFE and FAS-17, which diminishes the surface energy of the coatings [33,34]. The identification of Si (2.21 wt%) validates the integration of SiO_2_ nanoparticles into the composite coatings.

The EDS elemental images (Figure 2e,f) indicate that C, O, F, and Si are evenly distributed throughout the entire coatings surface. The consistent distribution of Si indicates that the SiO_2_ nanoparticles are well-integrated with the PTFE particles and the ER/FR binder matrix, thereby averting significant agglomeration. These results, in conjunction with SEM images, unequivocally indicate that PTFE/SiO_2_–ER/FR composite coatings featuring the hierarchical roughness structure and chemical composition are effectively prepared using the spray-coating method.

### 3.2. UV Irradiation and Freeze–Thaw Durability of the PTFE/SiO_2_–ER/FR Superhydrophobic Coatings

#### 3.2.1. UV Irradiation Durability

**Figure 3:**
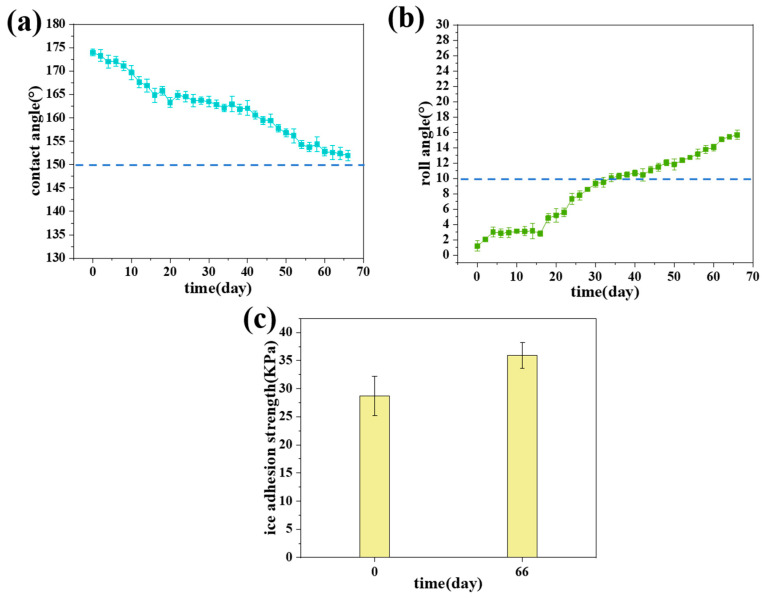
Effect of UV irradiation on the wettability and ice adhesion strength of the PTFE/SiO_2_–ER/FR coated glass slides. (**a**,**b**) Contact angle and roll angle evolution over time; (**c**) ice adhesion strength before and after 66 days of UV irradiation.

The UV durability of the PTFE/SiO_2_–ER/FR composite coatings was evaluated by tracking their surface wettability and ice adhesion strength over time (Figure 3). All contact angle and roll angle tests were performed at 26 °C with 55% RH. Ice adhesion strength tests were conducted at −10 °C with 55% RH. Before UV irradiation, the contact angle of the PTFE/SiO_2_–ER/FR composite coatings was 173.9°, whereas the roll angle was 1.2°. The coatings demonstrate a good contact angle and a reduced roll angle compared to previously documented PTFE-ER/FR coatings [35]. With an increase in UV irradiation duration, the contact angle of the coatings exhibits a progressive decline. After 66 days of UV irradiation, the contact angle persists at 151.9° (Figure 3a), demonstrating that the coatings retain their superhydrophobic performance. Moreover, the roll angle remains below 10° until 34 days post-irradiation (Figure 3b). The ice adhesion strength increases slightly from 28.7 kPa to 35.9 kPa (Figure 3c). These results confirm good UV durability of the PTFE/SiO_2_–ER/FR composite coatings.

**Figure 4:**
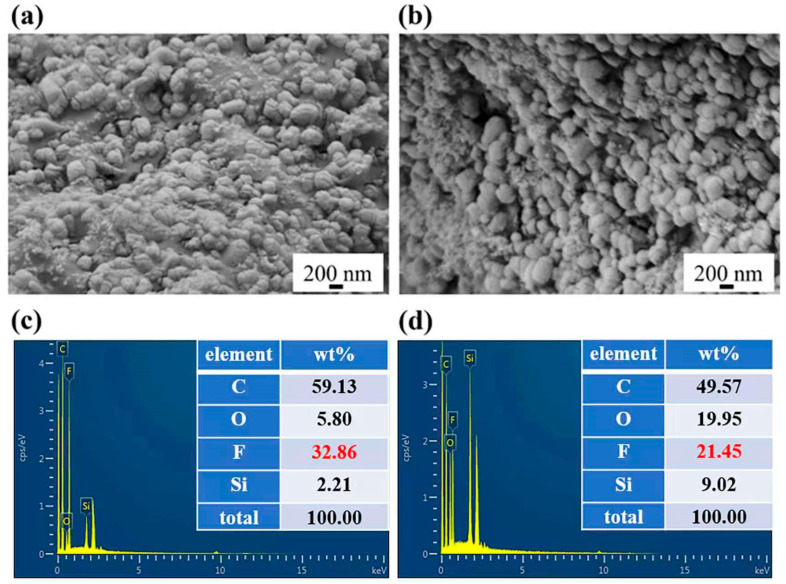
SEM images (**a**,**b**) and EDS images (**c**,**d**) of the PTFE/SiO_2_–ER/FR superhydrophobic coating before and after UV radiation.

The structural and chemical stability of the coatings after long-term UV irradiation were verified by SEM and EDS (Figure 4). The SEM images of the PTFE/SiO_2_–ER/FR superhydrophobic coatings prior to (Figure 4a) and subsequent to (Figure 4b) UV irradiation are observed in the micro–nano hierarchical structure, which remains relatively complete, suggesting that UV irradiation has a limited influence on the micro–nano structure. The initial fluorine concentration of the PTFE/SiO_2_–ER/FR superhydrophobic coatings is 32.86 wt% (Figure 4c). However, after 66 days of UV irradiation, the surface fluorine concentration diminishes to 21.45 wt% (Figure 4d), a decrease of 11.41 percentage points. A reduction in surface fluorine content may elevate surface free energy, therefore diminishing hydrophobicity and augmenting the coatings’ ice adhesion strength.

#### 3.2.2. Freeze–Thaw Durability

**Figure 5:**
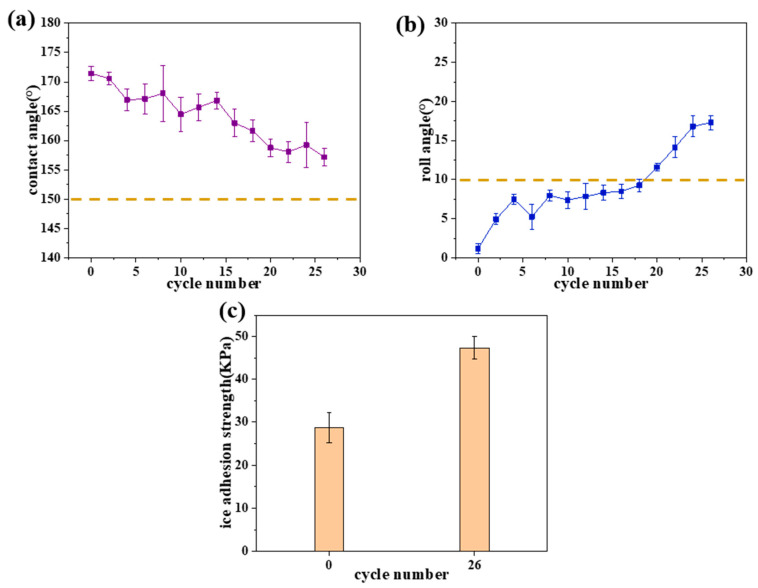
Effect of freeze–thaw cycle number on the wettability and ice adhesion strength of the PTFE/SiO_2_–ER/FR coated glass slides. (**a**,**b**) Contact angle and roll angle evolution over cycle number; (**c**) ice adhesion strength before and after 26 freeze–thaw cycles.

The freeze–thaw durability of the PTFE/SiO_2_–ER/FR composite coatings was evaluated by tracking their surface wettability and ice adhesion strength over cycle number (Figure 5). All contact angle and roll angle tests were performed at 26 °C with 55% RH. Ice adhesion strength tests were conducted at −10 °C with 55% RH. With the increase in freeze–thaw cycles, the contact angle of the coatings shows a progressive decline (Figure 5a). After 26 freeze–thaw cycles, the contact angle remains at around 157°, demonstrating that the coatings retain the superhydrophobic performance. Moreover, after 18 freeze–thaw cycles, the roll angle persists below 10° (Figure 5b). The ice adhesion strength marginally rises to 47.4 kPa (Figure 5c). These results confirm the good freeze–thaw durability of the PTFE/SiO_2_–ER/FR composite coatings.

**Figure 6:**
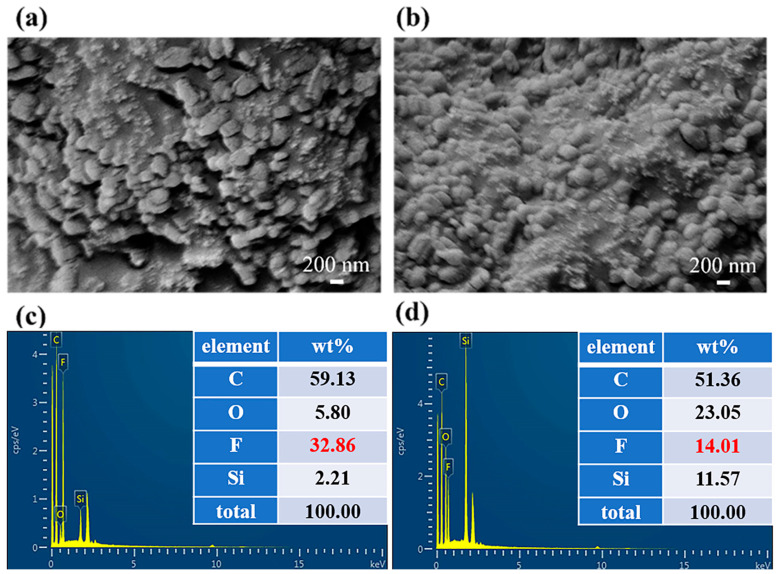
SEM images (**a**,**b**) and EDS images (**c**,**d**) of the PTFE/SiO_2_–ER/FR superhydrophobic coating before and after freeze–thaw cycles.

The structural and chemical stability of the coating after repeated freeze–thaw cycles were verified by SEM and EDS (Figure 6). The SEM images of the PTFE/SiO_2_–ER/FR superhydrophobic coatings prior to (Figure 6a) and subsequent to (Figure 6b) freeze–thaw cycles indicate that the micro–nano rough structure of the coatings is maintained after repeated freeze–thaw cycles. This performance may be ascribed to the resilient micro–nano structure created by SiO_2_, along with the cross-linked composite binder system established by ER and FR. After 26 freeze–thaw cycles, the surface fluorine content decreases from an initial 32.86 wt% (Figure 6c) to 14.01 wt% (Figure 6d). This may suggest that the low-surface-energy components on the coated surface diminish progressively with an increase in freeze–thaw cycles.

### 3.3. Anti-Icing and De-Icing Performance of the Coating in the Natural Conditions

The hydrophobicity and droplet beading behavior of glass insulators coated with a PTFE/SiO_2_–ER/FR superhydrophobic coating were assessed under natural field conditions.

**Figure 7:**
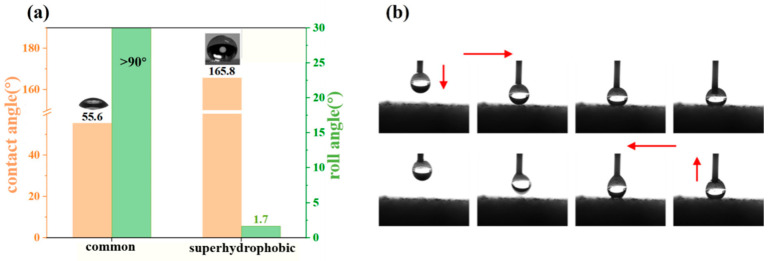
(**a**) Contact angle and roll angle of superhydrophobic and common insulators. (**b**) Water droplet adhesion image on the superhydrophobic insulators’ surface.

The wettability of the PTFE/SiO_2_–ER/FR coated glass insulators was evaluated under natural conditions (Figure 7). The contact angle on the surface of common insulators is 55.6°, accompanied by a roll angle beyond 90° (Figure 7a), signifying a clearly hydrophilic condition. The contact angle on the superhydrophobic insulators attains 165.8°, accompanied by a roll angle of 1.7° (Figure 7a). The water droplet does not attach to the surface of the hydrophobic performance on insulator surfaces (Figure 7b), hence confirming the coatings’ good hydrophobicity in natural conditions.

**Figure 8:**
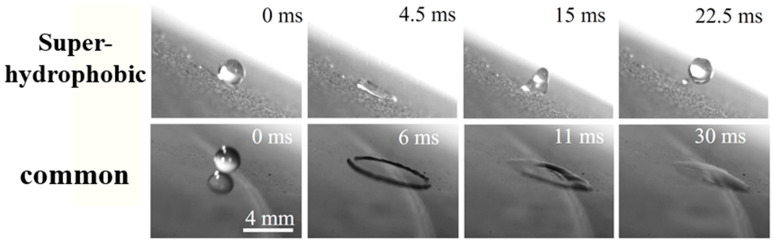
Bouncing behavior of single water droplet on superhydrophobic and common insulators’ surface under natural conditions.

The dynamic water repellency of the coated insulators is verified by observing droplet impact processes (Figure 8). On the superhydrophobic insulators’ surface (Figure 8, top row), the water droplet achieves its maximum spread area at 4.5 ms, exhibiting a spreading coefficient of 2.36. It subsequently separates from the surface for the first time at 15 ms, attains a peak rebound height of 1.99 mm at 22.5 ms, and rebounds a total of three times, with a contact duration of 15 ms. Upon striking the surface of common insulators (Figure 8, bottom row), the water droplet expands to its maximum area within 6 ms, exhibits a spreading coefficient of 3.12, and then adheres to the surface. The superhydrophobic insulators reach their peak spreading area in a shorter time, and the maximum spreading area is smaller. This suggests that the PTFE/SiO_2_–ER/FR coatings can efficiently repel the water droplet, reducing adherence and residence duration on the superhydrophobic insulators surface, thereby delaying ice nucleation and reducing ice accumulation.

**Figure 9:**
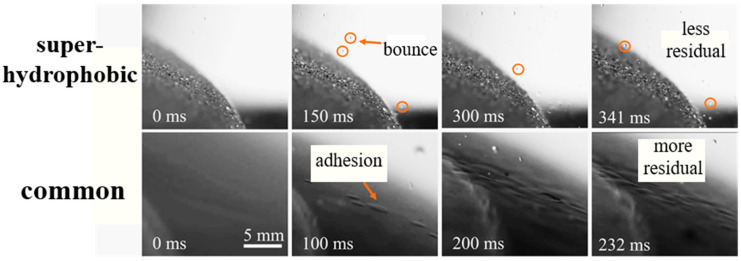
Bouncing behavior of sprayed water droplets on superhydrophobic and common insulators’ surface under natural conditions.

The hydrophobicity of the coated insulators under natural conditions was further verified by observing droplet dynamics (Figure 9). On superhydrophobic insulators (Figure 9, top row), droplets maintain a nearly spherical shape and rebound swiftly, demonstrating smaller surface stickiness that inhibits droplet retention. Droplets on common insulators disperse upon impact, leading to an extensive contact area (Figure 9, bottom row). The results demonstrate that the PTFE/SiO_2_–ER/FR coatings can restrain the formation of continuous water films.

**Figure 10:**
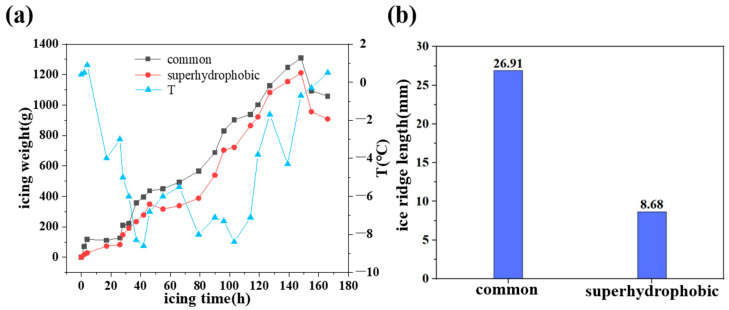
(**a**) Ice weight and (**b**) length of the longest ice ridge for superhydrophobic and common insulators under natural conditions.

The anti-icing and de-icing performance of the coated insulators is verified by monitoring ice weight and ridge growth under natural conditions (Figure 10). With the extension of icing time, the ambient temperature progressively decreases below 0 °C (Figure 10a, “T” line), oscillating between −2 °C and −8 °C during the 40–130 h interval. During testing period, the ice weight on the superhydrophobic insulators (Figure 10a, “superhydrophobic” line) consistently remains lower than that on the common insulators (Figure 10a, “common” line). During the last phases of the icing test, when the ambient temperature approaches 0 °C, the ice accumulation on both samples reduces. The ice weight on the superhydrophobic insulators is 909 g, representing a 14.2% reduction compared to the 1059 g measured for the common insulators. The good anti-icing and de-icing effect arises from the synergistic benefits of the superhydrophobic coating. Lower surface adhesion and micro- and nanoscale rough structure diminish the contact area of water droplets.

Moreover, the longest ice ridge length on the superhydrophobic insulators measures 8.68 mm, indicating a 67.7% decrease compared to 26.91 mm recorded on the common insulators (Figure 10b). This indicates that the coating can weaken the growth of ice ridges. The reduced ice adhesion strength of the PTFE/SiO_2_–ER/FR coatings compromises the robust adherence of ice crystals, rendering them susceptible to separation. This prevents bridging between adjacent insulators in the insulator string, thereby reducing the occurrence of electrical safety issues caused by ice flashovers.

## 4. Conclusions

PTFE/SiO_2_–ER/FR superhydrophobic coatings were prepared using the spraying method. The micro/nanostructure, surface wettability, anti-icing and de-icing performance, and environmental durability were thoroughly examined. The analysis focuses on the development of surface roughness, fluorine dispersion, and the progression of anti-icing and de-icing performance during UV irradiation and freeze–thaw cycles. Particular emphasis is placed on assessing the anti-icing and de-icing performance of the coated insulators in natural conditions. The main conclusions are summarized as follows:

(1) The PTFE/SiO_2_–ER/FR coatings featuring a micro/nanoscale hierarchical structure are successfully fabricated using the spraying method. SiO_2_ nanoparticles are uniformly distributed, occupying the interstices between PTFE particles, whereas the uniform distribution of fluorine elements significantly diminishes surface energy. This imparts the coatings with good superhydrophobicity, resulting in the contact angle of 173.9° and the roll angle of 1.2°.
(2) The composite coatings exhibit good environmental durability. Following 66 days of UV irradiation and 26 freeze–thaw cycles, the contact angle persists above 150° without obvious structural damage. Although aging causes partial loss of fluorine, the comprehensive hydrophobic, anti-icing and de-icing performance decreases slightly and remains at a satisfactory level.
(3) Under natural icing conditions, the PTFE/SiO_2_–ER/FR composite superhydrophobic coatings exhibit good anti-icing and de-icing performance. Water droplets bounce thrice on the coated insulators’ surface, attaining a rebound height of 1.99 mm, a spreading coefficient of 2.36, and a brief contact time of only 15 ms. Natural field icing studies indicate that the coated insulators exhibit reduced ice weight compared to common insulators. The ultimate ice accumulation diminishes by 14.2%, while the maximum ice ridge length is curtailed by 67.7%.

## Data Availability

The original contributions presented in the study are included in the article material. Further inquiries can be directed to the corresponding authors.
